# Nanoceria as Safe Contrast Agents for X-ray CT Imaging

**DOI:** 10.3390/nano13152208

**Published:** 2023-07-29

**Authors:** Ana García, Juan Antonio Cámara, Ana María Boullosa, Muriel F. Gustà, Laura Mondragón, Simó Schwartz, Eudald Casals, Ibane Abasolo, Neus G. Bastús, Víctor Puntes

**Affiliations:** 1 Design and Pharmacokinetics of Nanoparticles, CIBBIM-Nanomedicine, Vall d’Hebron Hospital Universitari, Vall d’Hebron Barcelona Hospital Campus, Passeig Vall d’Hebron 119-129, 08035 Barcelona, Spain; ana.garcia@vhir.org (A.G.); lmondragon@carrerasresearch.org (L.M.); 2 Preclinical Imaging Platform, Vall d’Hebron Institute of Research (VHIR), Universitat Autònoma de Barcelona (UAB), 08035 Barcelona, Spain; juanantonio.camaraserrano@ucsf.edu; 3 Clinical Biochemistry, Drug Delivery & Targeting (CB-DDT), Vall d’Hebron Hospital Universitari, Vall d’Hebron Barcelona Hospital Campus, Passeig Vall d’Hebron 119-129, 08035 Barcelona, Spain; ana.boullosa@vhir.org (A.M.B.); ibane.abasolo@vhir.org (I.A.); 4 Networking Research Centre on Bioengineering, Biomaterials and Nanomedicine (CIBER-BBN), 08034 Barcelona, Spain; muriel.freixanet@icn2.cat (M.F.G.); neus.bastus@icn2.cat (N.G.B.); 5 Institut Català de Nanociència i Nanotecnologia (ICN2), Consejo Superior de Investigaciones Científicas (CSIC), The Barcelona Institute of Science and Technology (BIST), 08036 Barcelona, Spain; 6 Servei de Bioquímica, Vall d’Hebron Hospital Universitari, Vall d’Hebron Barcelona Hospital Campus, Passeig Vall d’Hebron 119-129, 08035 Barcelona, Spain; 7 School of Biotechnology and Health Sciences, Wuyi University, Jiangmen 529020, China; wyuchemecm@126.com; 8 Institut Català de Recerca i Estudis Avançats, (ICREA), P. Lluis Companys 23, 08010 Barcelona, Spain

**Keywords:** nanoceria, CeO_2_ nanoparticles, X-ray CT imaging, reactive oxygen species scavengers

## Abstract

Cerium oxide nanoparticles (CeO_2_NPs) have exceptional catalytic properties, rendering them highly effective in removing excessive reactive oxygen species (ROS) from biological environments, which is crucial in safeguarding these environments against radiation-induced damage. Additionally, the Ce atom’s high Z number makes it an ideal candidate for utilisation as an X-ray imaging contrast agent. We herein show how the injection of albumin-stabilised 5 nm CeO_2_NPs into mice revealed substantial enhancement in X-ray contrast, reaching up to a tenfold increase at significantly lower concentrations than commercial or other proposed contrast agents. Remarkably, these NPs exhibited prolonged residence time within the target organs. Thus, upon injection into the tail vein, they exhibited efficient uptake by the liver and spleen, with 85% of the injected dose (%ID) recovered after 7 days. In the case of intratumoral administration, 99% ID of CeO_2_NPs remained within the tumour throughout the 7-day observation period, allowing for observation of disease dynamics. Mass spectrometry (ICP-MS) elemental analysis confirmed X-ray CT imaging observations.

## 1. Introduction

The precise enhanced medical imaging powered by contrast agents has become a universal diagnosis tool, especially in the case of techniques employing X-rays, which show high spatial resolution but relatively low sensitivity [1]. Currently, 40% of all X-ray medical images use contrast agents to improve diagnosis [2]. Iodine compounds are generally employed due to their biocompatibility and high Z number [3]; however, they are not exempt from limitations: ions are removed rapidly from the bloodstream making the imaging time window very short, so high doses are needed. These ions are filtered in the kidney, ultimately producing nephrotoxicity [4], the third more common cause of hospital-acquired acute renal injury [5].

Intense research has prompted for the last 20 years to develop new contrast agents that overcome these limitations and allow new imaging applications [6,7]. Among them, high Z number nanoparticles (NPs) have been proposed since their altered biodistributions and longer residence times permit extending the available imaging window and reducing administration volumes [8], opening the opportunity to study disease dynamics [9]. Initially, NPs are captured by the liver and the spleen, allowing advanced imaging in these organs [10]. Remarkably, NPs biodistribution is sensitive to disease. Thus, NPs are also prone to maximise their accumulation in lymph nodes [11], solid tumours [12,13], and inflammation areas [14,15] via enhanced permeability and retention (EPR) effects [12,15,16]. Consequently, NPs containing heavy atoms have been employed to image hepatocellular carcinoma in the liver [17] and tumours in the lymph nodes [18,19] or the brain [20], where NPs biodistribution depends on size, surface charge, surface structure and the presence of targeting moieties [21], allowing for site-directed imaging [8,10]. Thus, several NPs have been studied as an alternative to the iodinated molecules, including Gold [22], Bismute [23] or Ytterbium [24] based NPs at 100 mg/mL, 75 mg/mL and 70 mg/mL, respectively. They all show good contrast efficiency. They are non-toxic and biocompatible.

Despite low doses being used during X-ray imaging, about 700 traditional images are taken for a CT 3D reconstruction which is not exempt from side effects, especially in children. Several studies have reported the induction of chromosome aberrations, changes in gene expression and a significant increase in γ-H2AX foci levels in patients, which is indicative of DNA damage [25,26]. X-ray radiation can induce DNA damage at the molecular level, together with the ionisation of water and the generation of a large amount of reactive oxygen species (ROS), which ultimately accounts for 40% to 60% of the produced damage [27,28]. Unfortunately, it has been reported that the use of contrast agents during CT scans increases the radiation dose and toxicity [29]. This becomes even more dramatic when metallic NPs can catalyse the generation of free radicals, further increasing indirect damage [30].

In the meantime, CeO_2_NPs have been extensively studied due to their radioprotective properties, degrading radiation-induced toxic free radicals (ROS) into innocuous species, which has been translated into protection from indirect radiative damage [31,32,33,34,35,36]. CeO_2_NPs have a high capacity to buffer electrons in redox environments [37,38], followed by the capture or release of oxygen. It means that CeO_2_NPs act as a free-radical scavenger of ROS molecules such as OH• and H_2_O_2_ [39]. Despite that Ce has a higher atomic number, Z number (58), than I (53), its potential as a CT contrast agent has been relatively unexplored [40]. Interestingly, recent studies have focused on the potential use of gadolinium-doped CeO_2_NPs as contrast agents for magnetic resonance imaging with high T1 relaxivity [41,42]. Our study demonstrates the significant benefits of CeO_2_NPs as a contrast agent for CT imaging, primarily due to their high X-ray attenuation, excellent biocompatibility, and radioprotective properties. Importantly, these CeO_2_NPs exhibit slow biodegradation into innocuous species and efficient excretion, further enhancing their safety profile [43,44]. This combination of properties positions CeO_2_NPs as a highly competitive contrast agent, offering improved safety for CT imaging.

## 2. Results and Discussion

**Figure 1:**
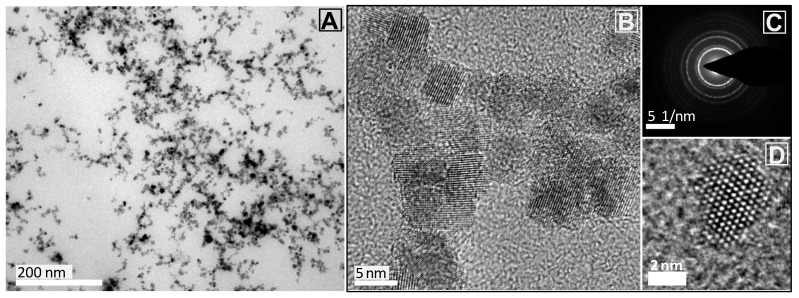
Characterisation of CeO_2_NPs conjugated with murine serum albumin (10 mg Ce/mL). (**A**) TEM image taken at 150,000× magnification showing CeO_2_NPs of 5.1 ± 1.4 nm average size; (**B**) HRTEM image taken at 450,000× magnification, (**C**) representative diffraction pattern; and (**D**) HRTEM of a single CeO_2_NPs.

Non-aggregated highly soluble CeO_2_NPs at 10 mg Ce/mL have been synthesised with an average size of 5.1 ± 1.4 nm (Figure 1). A size of ~5 nm was chosen to maximise their catalytic activity [45]. The as-synthesised NPs were immediately conjugated to an excess of Murine Serum Albumin (MSA), preventing them from aggregation. Conjugation with MSA increases biocompatibility and solubility in biological media [46], preventing rapid renal clearance [47] and avoiding aggregation in the highly ionic physiological media [48,49]. The colloidal solution presents a UV-Vis absorption peak at 290 nm, indicative of Ce^4+^, a ζ potential value of −28.2 ± 0.73 mV (conductivity 0.74 ± 0.02 mS/cm at pH 7.4) and a hydrodynamic diameter of 38.7 ± 1.9 nm (Figure S1). DLS analysis shows a monomodal peak stable over time, indicative of good colloidal stability and lack of large aggregates [50].

To evaluate the use of CeO_2_NPs as contrast agents for safe X-ray CT imaging, 200 μL of a CeO_2_NPs-MSA solution (10 mg Ce/mL vs. standard Iodine at 175 mg/mL concentrations) was injected intravenously through the tail vain into awakened female athymic nude mice of 7 weeks of age bearing xenografted subcutaneous sarcoma tumours of A-673 human cancer cells in the right rear flank. Five animals with tumour volumes above 700 mm^3^ were selected for the assay four weeks post-tumour inoculation. After NP administration, supervision of the animals and body weight measurement were performed every day, which did not indicate any symptoms of toxicity. Clinical observations included possible changes in skin, eyes, mucous membranes, alterations in respiratory pattern, behaviour, posture, response to handling or abnormal movements. Seven days after NP’s injection, animals were sacrificed. Blood, primary tumours and several organs -liver, spleen, kidneys, lung, heart and brain- were excised, weighed and kept at −20 °C for ICP-MS measurements. Obtained results (Table 1) show how CeO_2_NPs-MSA exhibited efficient uptake by the liver and spleen upon injection into the tail vein, with 85%ID being recovered after 7 days.

X-ray CT images were collected using a Quantum FX micro-CT instrument. A total of 512 projections were obtained in a 120 s/scan. The incident X-ray tube potential was set at 90 kVp and current at 200 μA. For the animals injected via the tail vein, X-ray CT images were taken before injection and at various representative post-administration times using a field of view (FOV) of 40 × 40 mm. The images were reconstructed with the Quantum FX software, based on Feldkamp’s method. Three-dimensional rendering videos of the intratumoral study were performed with Amide Image analysis [51], consisting of 3D radiodensity evaluation of different biological structures, including liver, spleen, kidney, sub-lumbar muscle and tumour tissue. Previous to each scan, animals were anaesthetised with Isofluorane. Once the scan was finished, animals were brought back to their cages for recovery.

**Figure 2:**
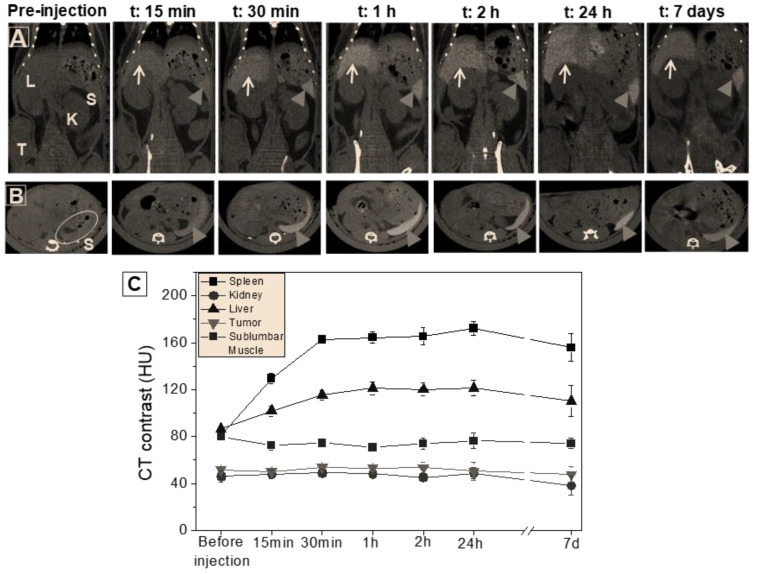
In vivo CT analysis of intravenous injection via tail vein of CeO_2_NPs-MSA solution (200 μL, 10 mg Ce/mL) in mice, at various representative times from 0 h (before injection) up to 7 days. (**A**) Serial CT coronal views showing the main regions of interest, i.e., liver, spleen, kidneys, and tumour, are indicated with their initials in the first image. Arrowheads point at the spleen, and the white arrows indicate the liver. (**B**) Corresponding CT axial views show the contrasting evolution of the spleen over time. (Other white areas correspond to the bone, and black regions correspond to air). (**C**) Temporal evolution of CT contrast density values (HU) of the tissues under study. Numerical HU values are provided in Table S1.

Combining the serial CT views and the quantitative analysis of contrast density allowed us to gain insights into the distribution and behaviour of CeO_2_NPs within the mice [52]. Figure 2 displays obtained results after the intravenous injection of CeO_2_NPs-MSA solution via the tail vein of mice. The mice were examined at various time points, ranging from 0 h (before injection) up to 7 days, allowing us to capture the temporal evolution of the NPs within the mice. In the serial CT coronal views, we identified the regions of interest: the liver, spleen, kidneys, and tumour (Figure 2A). These regions were labelled with their initials in the first image to facilitate clear identification. The spleen was specifically highlighted with arrowheads, while white arrows denoted the liver. Monitoring the contrasting evolution of the spleen over time was achieved through the observation of CT axial views (Figure 2B). In these images, the white areas correspond to the bone, while the black regions represent air. By analysing the changes in contrast density, we quantified the temporal evolution of CT contrast density values (HU) for the investigated tissues (Figure 2C). Detailed information on 3D rendering and specific HU values can be found in Figure S2 and Table S1.

**Figure 3:**
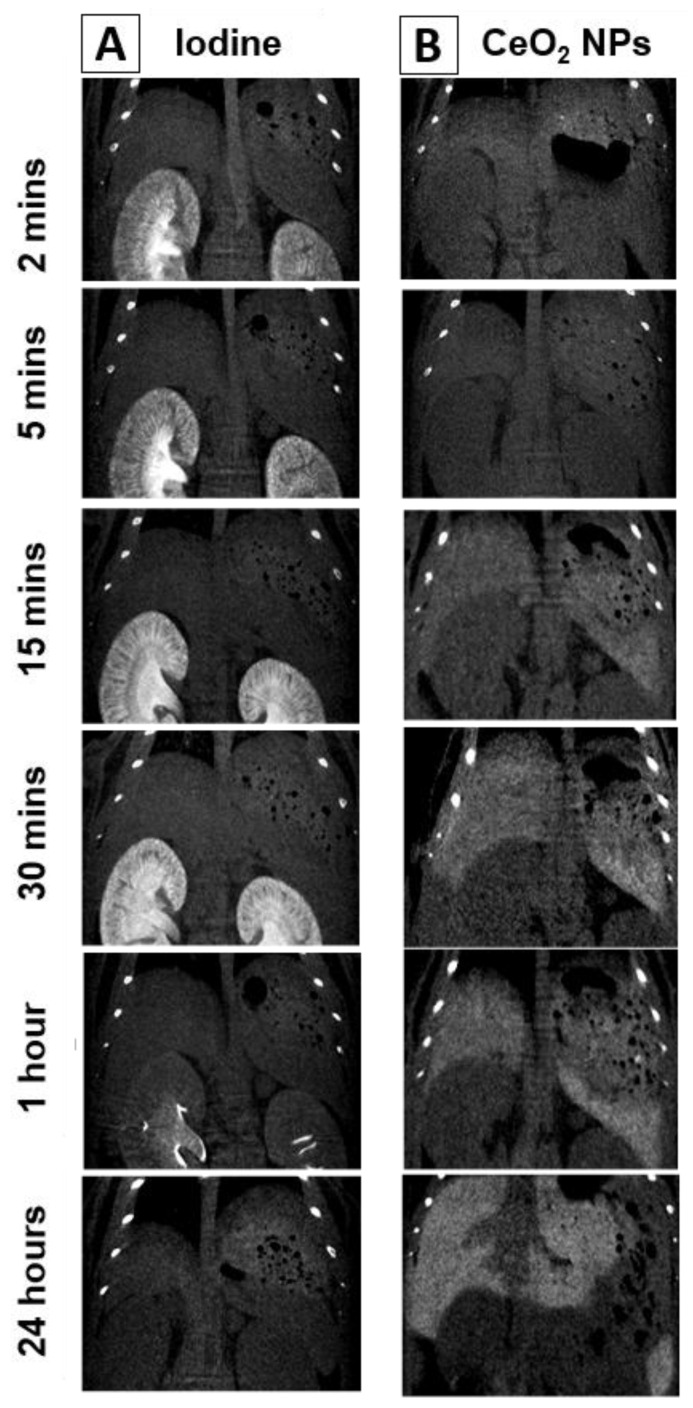
In vivo coronal CT images after intravenous injection of (**A**) the commercial iodine contrast agent Iopamidol^®^-370 (150 µL; 175 mg I/mL) and (**B**) CeO_2_NPs-MSA solution (200 μL, 10 mg Ce/mL) at various representative times, from 2 min post-injection up to 24 h.

Figure 3 presents a zoomed image of the liver, providing insights into the distribution of the NPs within the liver parenchyma. The image reveals that the NPs are homogeneously distributed within the liver tissue without significant accumulation around the blood vessels. These results indicate that NPs possess good solubility and dispersion in the organ, suggesting the absence of an immune defensive response against the CeO_2_NPs [53] following the non-immunogenic character of these NPs previously described in the literature [54,55]. In contrast, intravenous injection of the commercial iodine contrast agent Iopamidol^®^-370 leads to a fast accumulation in kidneys (5 min post-injection) and subsequent quick renal excretion, with almost no remaining signal at 24 h post-administration (Figure S3).

**Figure 4:**
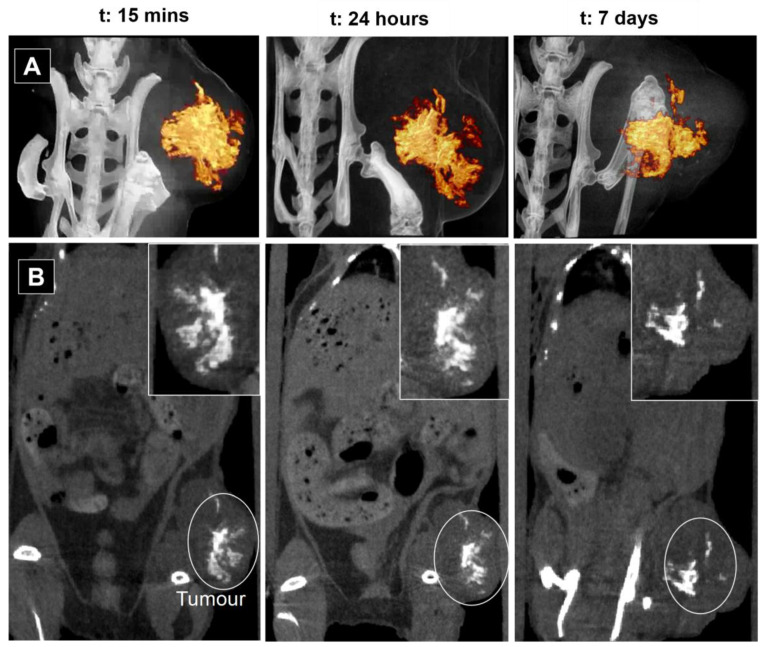
In vivo CT images after intratumoral injection of 70 μL of CeO_2_NPs-MSA solution (10 mg Ce/mL) in mice at various time intervals: 15 min, 24 h and 7 days. (**A**) Snapshot of the 3D rendering videos (videos are provided in Figure S4). (**B**) Coronal view of a 2D CT projection. A zoomed image of the tumoral area is shown in the insight of each image.

To investigate the effects of intratumoral administration, we administered 70 μL of CeO_2_NPs-MSA solution directly into the tumour. Figure 4 shows serial CT coronal view images of the mouse at 15 min, 24 h and 7 days post-injection. CT scans were performed with a FOV of 24 × 24 mm and 60 × 60 mm for the mouse injected intratumorally. A quantitative analysis of the 3D tomographic reconstruction is given in Table 2. The 3D rendering videos at the three representative times can be seen in Figure S4. A clear enhancement of contrast signal was observed in the tumour 15 min after injection (747.2 HU compared to 75.5 HU), remaining enhanced for the 7 days of measurement. This is in contrast with intratumoral injection of iodine compounds such as Iohexol, which disappears from the tumour in 4 h, while the nanoparticulate polymeric Iodine, Poly(iohexol), loses 50% of the contrast also at 4 h [56].

Long residence times of the contrast agent allow for observing the structural evolution of tumours. During the 7 days of observation, the tumour grew from 700 mm^3^ to 1500 mm^3^. Interestingly, contrast levels of the adjacent muscle remained at similar values over time, which indicates that CeO_2_NPs did not spread through nearby tissues [57].

ICP-MS analyses performed after animal sacrifice on day 7 confirmed the CT observations (Table S2). No trace of Ce was detected in the mouse organs (liver, spleen, kidneys, lungs, heart and brain) or blood plasma other than in the tumour. Tumour analysis yields a total Ce mass of 706.6 ± 35 µg, which corresponds to 98.9 ± 4.7%ID (ID: 714 ± 36 µg Ce), indicating that CeO_2_NPs-MSA remain inside the tumour for the 7 days. Analysing contrast volume and intensity as a function of time, we observe how the measured contrasted area gets compressed with increased contrast intensities as the tumour grows. This fact is attributed to the interstitial stress and fluid pressure within a growing tumour [58].

Mechanical stress is an important parameter that regulates tissue oxygenation, cancer cell proliferation [59] and drug delivery [60] during the progression of solid tumours. Heterogeneous mechanical solid stresses developed during tumour growth compress blood vessels, dramatically reducing the supply of oxygen and access to drugs in inner regions, promoting the formation of hypoxic and necrotic regions in the tumour [58]. Thus, CeO_2_NPs-MSA may be used as a long-lasting probe to analyse tumour evolution over time for disease progression prediction, especially in cases where there is a need to clearly distinguish between pseudoprogression and tumour progression, like in glioblastoma [61]. Accurately evaluating animal models mimicking human disease is vital when translating these studies into the clinic. Non-invasive imaging is a clinical standard and has enormous yet underexploited benefits for advanced medical imaging and research if one can repeatedly evaluate host response (inflammation, tissue remodelling) and disease progression needed to gain better insight into the dynamics of the pathology and treatment effects [9], especially with CeO_2_NPs which are radioprotective [31,32,33], can be made safe [62] and have been observed to slowly biodegrade [43,44].

CeO_2_NPs-MSA provide good contrast levels in vivo even when injected at comparatively low concentrations. Moreover, the well-known antioxidant properties of CeO_2_NPs (Figure S5) suppose an additional motivation for their clinical use, as they may help to mitigate the detrimental effects of the ionising radiation used in CT scans, especially in children.

## 3. Conclusions

In this study, non-aggregated colloidally stable CeO_2_NPs conjugated with murine serum albumin (MSA) were synthesised and administered intravenously to mice with xenografted subcutaneous sarcoma tumours. The distribution of the NPs was monitored using X-ray CT imaging. The results showed that the NPs were homogeneously distributed within the liver tissue without significant accumulation around blood vessels, indicating good solubility and dispersion. In contrast, the commercial iodine contrast agent Iopamidol^®^-370 showed fast accumulation in the kidneys and subsequent renal excretion.

Furthermore, intratumoral administration of CeO_2_NPs-MSA resulted in a clear enhancement of contrast signal in the tumour, which remained enhanced for the entire 7-day observation period and the long residence times of the contrast agent allowed for observing structural changes in the tumour over time. Analysis of the tumour and other organs confirmed the presence of Ce only in the tumour, indicating that the CeO_2_NPs-MSA remained inside the tumour for the entire 7-day period. This prolonged retention within the tumour can provide valuable insights into tumour growth and dynamics.

Overall, the findings of this study demonstrate the potential of CeO_2_NPs-MSA as a contrast agent for safer CT scans, offering prolonged residence times, good solubility, and dispersion. Further research and development in this area could lead to the translation of CeO_2_NPs-MSA into clinical applications for enhanced medical imaging and improved diagnosis of diseases.

## 4. Materials and Methods

### 4.1. Materials

Cerium (III) nitrate hexahydrate (Ce(NO_3_)_3_·6H_2_O), tetramethylammonium hydroxide (TMAOH; 1M), and mouse serum albumin (MSA) (research grade, sterile filtered) were purchased from Sigma–Aldrich (Saint Louis, MO, USA). All reagents were used as received. Milli-Q water was used in the preparation of all solutions.

### 4.2. CeO_2_NPs Synthesis

CeO_2_NPs of around 5 nm were synthesised by the chemical precipitation of cerium (III) nitrate hexahydrate (Ce(NO_3_)_3_·6H_2_O) in a basic aqueous solution [48]. A total of 37.5 mM of cerium (III) nitrate hexahydrate was dissolved in 50 mL Milli-Q water at room temperature. A total of 50 mL of tetramethylammonium hydroxide (TMAOH) solution (44 mM) was added slowly at room temperature (RT) under vigorous stirring. The mixture was left under soft stirring for about 30 min. During the first minutes, the solution is colourless, then turns progressively brownish. NPs were purified by centrifugation (20,000× *g*, 45 min, RT), and the resultant reddish pellet was resuspended in 100mL aqueous solution of 1 mM TMAOH. As determined by the Xylenol Orange test (see below), the final Ce concentration was 1.06 ± 0.05 mg/mL (2.6 × 10^15^ NPs/mL). Before concentration, as-synthesised CeO_2_NPs were first conjugated with MSA. A total of 10 mg of MSA was dissolved in 1 mL of PB 50 mM and kept undisturbed (without stirring) in the fridge until its complete dissolution. Then, 10 mL of CeO_2_NPs were added dropwise to the protein solution while applying mild stirring. The sample was kept in the fridge for 24 h to allow the complete adsorption of MSA molecules onto the NP surface. The colloidal solution was then concentrated through 5 cycles of centrifugal filtration (Molecular weight cut-off 30 kDa, Millipore Amico Ultra) at 2500× *g* for 5 min, yielding 1ml of CeO_2_NPs-MSA at 10.2 ± 0.5 mg Ce/mL (75.8 mM Ce, 2.6 × 10^16^ NPs/mL), as determined by ICP-MS and Xylenol-orange test. Both the synthesis and concentration of NP were performed under sterile conditions.

### 4.3. Characterisation

The resultant NPs were characterised using a combination of techniques.

#### 4.3.1. UV-Vis Spectroscopy

UV-Vis spectra were acquired with a Cary 60spectrophotometer (Agilent Technologies, Santa Clara, CA, USA) in the 200–800 nm range using 1.5 mL plastic cuvettes. 

#### 4.3.2. Dynamic Light Scattering (DLS) and ζ-Potential

Malvern ZetaSizer Nano ZS instrument operating with a light source wavelength of 532 nm and a fixed scattering angle of 173° was used to determine the colloidal stability of the samples, the hydrodynamic diameter and ζ-potential value. Three independent measurements were performed. 

#### 4.3.3. Transmission Electron Microscopy

Samples were prepared by drop casting 10 µL of the sample onto carbon-coated copper grids and left to dry at room temperature. TEM images were acquired with a JEOL 1010 electron microscope (Jeol, Tokyo, Japan) operated at 80 kV accelerating voltage. HRTEM images were acquired with an FEI Tecnai G2 F20, operated at 200kV. NPs size distribution was determined using ImageJ software (National Institutes of Health, Bethesda, Rockville, MD, USA) by analysing more than 500 particles. 

#### 4.3.4. Ce^3+^ Concentration by Xylenol Orange Test

The test performed is based on the protocol developed by Tonosaki et al. [58]; orange forms a complex with free Ce^3+^ ions in a ratio of 1:1, which shows an absorption maximum at 575 nm. Briefly, 10 µL of the sample was added to a vial containing 700 μL of Xylenol Orange 1 mM, 3000 μL of acetate buffer (pH = 6.0) and 1290 μL of MilliQ water. The content of Ce^3+^ in an unknown sample was determined through the calibration graph obtained previously for 0–40 μg/mL of Ce^3+^. Here, the supernatant of the reaction was analysed.

### 4.4. In Vivo Assays

Female athymic nude mice (Janvier, Le Genest Saint Isle Saint Berthevin, France and Envigo Crs. SA, Barcelona, Spain) were kept in pathogen-free conditions and used at 7 weeks of age. Animal care was handled following the Guide for the Care and Use of Laboratory Animals of the Vall d’Hebron University Hospital Animal Facility, and the experimental procedures were approved by the Animal Experimentation Ethical Committee at the institution. In vivo studies were performed by the ICTS “NANBIOSIS” at the CIBER-BBN’s in vivo Experimental Platform of the Functional Validation and Preclinical Research (FVPR) area (https://www.nanbiosis.es/equipments/, accessed on 26 July 2023) (Barcelona, Spain).

A-673-Fluc cells (2 × 10^6^) suspended in 200 μL of cell culture media were subcutaneously inoculated in the right rear flank of each animal. Tumour growth was monitored twice a week by conventional calliper measurements (D × d^2^/2, where D is the major diameter and d is the minor diameter). Five animals with tumour volumes above 700 mm^3^ were selected for the assay four weeks post-tumour inoculation. In two of them, 200 µL of CeO_2_NPs solution was administered intravenously through the tail vein in awakened animals. For comparison, another two mice were injected intravenously with a commercial iodine contrast agent (Iopamidol^®^-370, 150 µL of 175 µg I/mL). The fifth animal was anaesthetised with 1.5% isofluorane (Forane, Baxter, Deerfield, IL, USA) and administered intratumorally with 70 µL of the exact solution of CeO_2_NPs-MSA. After NP administration, supervision of the animals and body weight measurement were performed daily, indicating no symptoms of toxicity. Clinical observations included possible changes in skin, eyes, mucous membranes, alterations in respiratory pattern, behaviour, posture, response to handling, and abnormal movements. Seven days after NP injection, animals were sacrificed. Blood plasma, primary tumours and several organs (liver, spleen, kidneys, lung, heart, brain and the tumour) were excised, weighed and kept at −20 °C for ICP-MS measurements.

The procedures for animal handling, humanitarian endpoint and animal euthanasia described in this project have been approved by the VHIR’s Animal Experimentation Ethics Committee (internal reference 38/17).

### 4.5. ICP-MS Analysis

Ce concentration in the CeO_2_NPs-MSA solution, blood plasma, tumour and various organs were determined by Inductively Coupled Plasma-Mass Spectrometry (Agilent, 7500ce). Plasma samples were diluted in a solution of EDTA 0.05% (*p*/*v*) and NH_3_ 0.5% (*v*/*v*). The tumour and organs were dissolved in HNO_3_ concentrated (Merck; p.a.) and heated in a microwave digestion oven (Milestone, Ultrawave). In parallel, a control sample was also digested. The resultant digestions were diluted with HNO_3_ 1% (*v*/*v*) before being injected into the ICP-MS instrument.

### 4.6. CT Imaging

X-ray CT images were collected using a Quantum FX micro-CT instrument (Perkin Elmer, Waltham, MA, USA) (see Supplementary Figure S6). A total of 512 projections were obtained in a 120 s/scan at a maximal resolution of 0.05mm, incident X-ray tube potential was set at 90 kVp and current at 200 μA. For the animals injected via tail vein, X-ray CT images were taken before injection and at various representative post-administration times (15 min, 30 min, 1 h, 2 h, 24 h and 7 days) using a field of view (FOV) of 40 × 40 mm. CT scans were performed at 15 min, 24 h and 7 days post-injection with a FOV of 24 × 24 mm and 60 × 60 mm for the mouse injected intratumorally. Reconstruction of the studies was performed with the Quantum FX software, based on Feldkamp’s method. Three-dimensional rendering videos of the intratumoral study were performed with Amide [45]. Imaging data were analysed at the Preclinical Imaging Platform from the Vall d’Hebron Research Institute. Image analysis consisted of 3D radiodensity evaluation of different biological structures, including liver, spleen, kidney, sub-lumbar muscle and tumour tissue. In each of these structures, at least four different regions of interest (ROIs) were selected, and the average density was obtained. CT attenuation values are given in the standard Hounsfield Units (HU) by calibration with water (HU = 0) and air (HU = −1000). The CT equipment is not normalised; therefore, the density value of the sub-lumbar muscle is used as a reference.

Before each scan, animals were anaesthetised with Isofluorane (5% during the induction phase, 2% during maintenance). Air flow was set to 0.8 L/min. Once the scan was finished, animals were brought back to their cages for recovery. All the procedures were performed following the institutional ethic committee.

## Figures and Tables

**Table 1:** ICP-MS analysis of Ce mass in mice organs (7 days post-i.v. injection), the weight of organs, total Ce mass in each organ and the corresponding %ID (2040 ± 98 mg Ce).

	Ce (μg/Organ g)	Organ Weight (g)	Total Ce (μg)	% Injected Dose (%ID)
Spleen	1746 ± 87	0.42	741 ± 42.3	36.3 ± 2.1%
Liver	579 ± 29	1.67	969 ± 49.1	48.1 ± 2.4%
Kidney	3.2 ± 0.2	0.38	1.22 ± 0.06	0.06%
Lung	102 ± 5.1	0.16	16.7 ± 0.84	0.8 ± 0.04%
Heart	2.3 ± 0.12	0.14	0.32 ± 0.02	0.02%
Brain	0.05 ± 0.003	0.41	0.02 ± 0.001	<0.01%
Tumour	0.60 ± 0.03	1.40	0.84 ± 0.04	0.04%
Plasma	4.41 ± 0.22	2.65	11.68± 0.63	6.01%
Total Ce (μg/g):			1754 ± 92.4	91.4 ± 4.3%

**Table 2:** CT contrast analysis (HU and volume) of the tumoral region after intratumoral injection of CeO_2_NPs-MSA solution (70 μL, 10 mg Ce/mL).

	Muscle CT Value (HU)	Non-Contrasted Tumour Tissue CT Value (HU)	Ce Contrast Agent CT Value (HU)	Ce Contrast Agent Vol (mm^3^)	Tumour Vol (mm^3^)
15 min	75.5 ± 3.9	75.4 ± 5.7	747.2 ± 44.9	50.2	1027.1
24 h	76.7 ± 4.1	73.9 ± 2.6	898.3 ± 35.9	42.3	1157.6
7 days	78.1 ± 1.0	72.6 ± 1.2	969.9 ± 38.7	37.4	1525.1

## Data Availability

Data available on request from the authors.

## Supplementary Materials

The following supporting information can be downloaded at: https://www.mdpi.com/article/10.3390/nano13152208/s1, Figure S1. Characterization of CeO2NPs conjugated with murine serum albumin; Table S1: CT contrast values (H.U.) corresponding to Figure 2 of the main article; Figure S2. Examples of in vivo 3D rendering videos before injection of CeO2NPs and 2 h after intravenous injection; Figure S3. CT coronal images 5 min and 24 h after intravenous injection of CeO2NPs-MSA; Figure S4. In vivo 3D rendering videos and their corresponding snapshots were recorded at 15 min, 24h and 7 days post-intratumoral injection; Table S2. ICP-MS analysis of organs after intratumoral injection; Figure S5. CeO2NPs-albumin conjugated and naked CeO2NPs capacity to scavenge hydrogen peroxide. Figure S6. (Left) Mouse anesthetized before a CT scans. (Right) Mouse placed in the plate of the Quantum FX micro-CT instrument (Perkin Elmer, Waltham, MA). References [57,63] are cited in the supplementary materials.

## References

[1] Kalender W.A. (2006). X-ray computed tomography. Phys. Med. Biol..

[2] Goldman L.W. (2007). Principles of CT: Radiation dose and image quality. J. Nucl. Med. Technol..

[3] deKrafft K.E., Xie Z., Cao G., Tran S., Ma L., Zhou O.Z., Lin W. (2009). Iodinated nanoscale coordination polymers as potential contrast agents for computed tomography. Angew. Chem. Int. Ed. Engl..

[4] Bottinor W., Polkampally P., Jovin I. (2013). Adverse reactions to iodinated contrast media. Int. J. Angiol..

[5] Mohammed N.M., Mahfouz A., Achkar K., Rafie I.M., Hajar R. (2013). Contrast-induced Nephropathy. Heart Views.

[6] Jakhmola A., Anton N., Vandamme T.F. (2012). Inorganic nanoparticles based contrast agents for X-ray computed tomography. Adv. Healthc. Mater..

[7] Lusic H., Grinstaff M.W. (2013). X-ray-computed tomography contrast agents. Chem. Rev..

[8] Shilo M., Reuveni T., Motiei M., Popovtzer R. (2012). Nanoparticles as computed tomography contrast agents: Current status and future perspectives. Nanomedicine.

[9] De Langhe E., Vande Velde G., Hostens J., Himmelreich U., Nemery B., Luyten F.P., Vanoirbeek J., Lories R.J. (2012). Quantification of lung fibrosis and emphysema in mice using automated micro-computed tomography. PLoS ONE.

[10] Moghimi S.M., Hunter A.C., Murray J.C. (2001). Long-circulating and target-specific nanoparticles: Theory to practice. Pharmacol. Rev..

[11] Reddy S.T., Rehor A., Schmoekel H.G., Hubbell J.A., Swartz M.A. (2006). In vivo targeting of dendritic cells in lymph nodes with poly(propylene sulfide) nanoparticles. J. Control. Release.

[12] Maeda H., Tsukigawa K., Fang J. (2016). A Retrospective 30 Years After Discovery of the Enhanced Permeability and Retention Effect of Solid Tumors: Next-Generation Chemotherapeutics and Photodynamic Therapy--Problems, Solutions, and Prospects. Microcirculation.

[13] Matsumoto Y., Nichols J.W., Toh K., Nomoto T., Cabral H., Miura Y., Christie R.J., Yamada N., Ogura T., Kano M.R. (2016). Vascular bursts enhance permeability of tumour blood vessels and improve nanoparticle delivery. Nat. Nanotechnol..

[14] Jin K., Luo Z., Zhang B., Pang Z. (2018). Biomimetic nanoparticles for inflammation targeting. Acta Pharm. Sin. B.

[15] Viscido A., Capannolo A., Latella G., Caprilli R., Frieri G. (2014). Nanotechnology in the treatment of inflammatory bowel diseases. J. Crohns Colitis.

[16] Maeda H. (2015). Toward a full understanding of the EPR effect in primary and metastatic tumors as well as issues related to its heterogeneity. Adv. Drug Deliv. Rev..

[17] Rand D., Ortiz V., Liu Y., Derdak Z., Wands J.R., Tatíček M., Rose-Petruck C. (2011). Nanomaterials for X-ray imaging: Gold nanoparticle enhancement of X-ray scatter imaging of hepatocellular carcinoma. Nano Lett..

[18] Oh M.H., Lee N., Kim H., Park S.P., Piao Y., Lee J., Jun S.W., Moon W.K., Choi S.H., Hyeon T. (2011). Large-scale synthesis of bioinert tantalum oxide nanoparticles for X-ray computed tomography imaging and bimodal image-guided sentinel lymph node mapping. J. Am. Chem. Soc..

[19] Pons T., Pic E., Lequeux N., Cassette E., Bezdetnaya L., Guillemin F., Marchal F., Dubertret B. (2010). Cadmium-free CuInS2/ZnS quantum dots for sentinel lymph node imaging with reduced toxicity. ACS Nano.

[20] Bobyk L., Edouard M., Deman P., Vautrin M., Pernet-Gallay K., Delaroche J., Adam J.F., Estève F., Ravanat J.L., Elleaume H. (2013). Photoactivation of gold nanoparticles for glioma treatment. Nanomedicine.

[21] Cimini A., D’Angelo B., Das S., Gentile R., Benedetti E., Singh V., Monaco A.M., Santucci S., Seal S. (2012). Antibody-conjugated PEGylated cerium oxide nanoparticles for specific targeting of Aβ aggregates modulate neuronal survival pathways. Acta Biomater..

[22] Hainfeld J.F., Slatkin D.N., Focella T.M., Smilowitz H.M. (2006). Gold nanoparticles: A new X-ray contrast agent. Br. J. Radiol..

[23] Bonitatibus P.J., Torres A.S., Goddard G.D., FitzGerald P.F., Kulkarni A.M. (2010). Synthesis, characterisation, and computed tomography imaging of a tantalum oxide nanoparticle imaging agent. Chem. Commun..

[24] Liu Y., Ai K., Liu J., Yuan Q., He Y., Lu L. (2012). A high-performance ytterbium-based nanoparticulate contrast agent for in vivo X-ray computed tomography imaging. Angew. Chem. Int. Ed. Engl..

[25] Shi L., Tashiro S. (2018). Estimation of the effects of medical diagnostic radiation exposure based on DNA damage. J. Radiat. Res..

[26] Sokolov M., Neumann R. (2016). Global Gene Expression Alterations as a Crucial Constituent of Human Cell Response to Low Doses of Ionizing Radiation Exposure. Int. J. Mol. Sci..

[27] Sanche L. (2005). Low energy electron-driven damage in biomolecules. Eur. Phys. J. D-At. Mol. Opt. Plasma Phys..

[28] Sonntag C. (2006). Free-Radical-Induced DNA Damage and Its Repair: A Chemical Perspective.

[29] Wang L., Li Q., Wang X.M., Hao G.Y., Jie B., Hu S., Hu C.H. (2017). Enhanced radiation damage caused by iodinated contrast agents during CT examination. Eur. J. Radiol..

[30] McMahon S.J., Hyland W.B., Muir M.F., Coulter J.A., Jain S., Butterworth K.T., Schettino G., Dickson G.R., Hounsell A.R., O’Sullivan J.M. (2011). Biological consequences of nanoscale energy deposition near irradiated heavy atom nanoparticles. Sci. Rep..

[31] Colon J., Herrera L., Smith J., Patil S., Komanski C., Kupelian P., Seal S., Jenkins D.W., Baker C.H. (2009). Protection from radiation-induced pneumonitis using cerium oxide nanoparticles. Nanomedicine.

[32] Colon J., Hsieh N., Ferguson A., Kupelian P., Seal S., Jenkins D.W., Baker C.H. (2010). Cerium oxide nanoparticles protect gastrointestinal epithelium from radiation-induced damage by reduction of reactive oxygen species and upregulation of superoxide dismutase 2. Nanomedicine.

[33] Tarnuzzer R.W., Colon J., Patil S., Seal S. (2005). Vacancy engineered ceria nanostructures for protection from radiation-induced cellular damage. Nano Lett..

[34] Popov A.L., Zaichkina S.I., Popova N.R., Rozanova O.M., Romanchenko S.P., Ivanova O.S., Smirnov A.A., Mironova E.V., Selezneva I.I., Ivanov V.K. (2016). Radioprotective effects of ultra-small citrate-stabilised cerium oxide nanoparticles in vitro and in vivo. RSC Adv..

[35] Casals E., Zeng M., Parra-Robert M., Fernández-Varo G., Morales-Ruiz M., Jiménez W., Puntes V., Casals G. (2020). Cerium Oxide Nanoparticles: Cerium Oxide Nanoparticles: Advances in Biodistribution, Toxicity, and Preclinical Exploration (Small 20/2020). Small.

[36] Kadivar F., Haddadi G., Mosleh-Shirazi M.A., Khajeh F., Tavasoli A. (2020). Protection effect of cerium oxide nanoparticles against radiation-induced acute lung injuries in rats. Rep. Pract. Oncol. Radiother..

[37] Esch F., Fabris S., Zhou L., Montini T., Africh C., Fornasiero P., Comelli G., Rosei R. (2005). Electron localisation determines defect formation on ceria substrates. Science.

[38] Karakoti A.S., Singh S., Kumar A., Malinska M., Kuchibhatla S.V., Wozniak K., Self W.T., Seal S. (2009). PEGylated nanoceria as radical scavenger with tunable redox chemistry. J. Am. Chem. Soc..

[39] Chen J., Patil S., Seal S., McGinnis J.F. (2006). Rare earth nanoparticles prevent retinal degeneration induced by intracellular peroxides. Nat. Nanotechnol..

[40] Naha P.C., Hsu J.C., Kim J., Shah S., Bouché M., Si-Mohamed S., Rosario-Berrios D.N., Douek P., Hajfathalian M., Yasini P. (2020). Dextran-Coated Cerium Oxide Nanoparticles: A Computed Tomography Contrast Agent for Imaging the Gastrointestinal Tract and Inflammatory Bowel Disease. ACS Nano.

[41] Popov A.L., Abakumov M.A., Savintseva I.V., Ermakov A.M., Popova N.R., Ivanova O.S., Kolmanovich D.D., Baranchikov A.E., Ivanov V.K. (2021). Biocompatible dextran-coated gadolinium-doped cerium oxide nanoparticles as MRI contrast agents with high T1 relaxivity and selective cytotoxicity to cancer cells. J. Mater. Chem. B.

[42] Eriksson P., Truong A.H.T., Brommesson C., du Rietz A., Kokil G.R., Boyd R.D., Hu Z., Dang T.T., Persson P.O.A., Uvdal K. (2022). Cerium Oxide Nanoparticles with Entrapped Gadolinium for High T1 Relaxivity and ROS-Scavenging Purposes. ACS Omega.

[43] Hirst S.M., Karakoti A., Singh S., Self W., Tyler R., Seal S., Reilly C.M. (2013). Bio-distribution and in vivo antioxidant effects of cerium oxide nanoparticles in mice. Environ. Toxicol..

[44] Muhammad F., Wang A., Qi W., Zhang S., Zhu G. (2014). Intracellular antioxidants dissolve man-made antioxidant nanoparticles: Using redox vulnerability of nanoceria to develop a responsive drug delivery system. ACS Appl. Mater. Interfaces.

[45] Cafun J.D., Kvashnina K.O., Casals E., Puntes V.F., Glatzel P. (2013). Absence of Ce^3+^ sites in chemically active colloidal ceria nanoparticles. ACS Nano.

[46] Caputo F., Mameli M., Sienkiewicz A., Licoccia S., Stellacci F., Ghibelli L., Traversa E. (2017). A novel synthetic approach of cerium oxide nanoparticles with improved biomedical activity. Sci. Rep..

[47] Choi H.S., Liu W., Misra P., Tanaka E., Zimmer J.P., Itty Ipe B., Bawendi M.G., Frangioni J.V. (2007). Renal clearance of quantum dots. Nat. Biotechnol..

[48] Bastus N.G., Casals E., Vázquez-Campos S., Puntes V. (2008). Reactivity of engineered inorganic nanoparticles and carbon nanostructures in biological media. Nanotoxicology.

[49] Casals E., Pfaller T., Duschl A., Oostingh G.J., Puntes V. (2010). Time evolution of the nanoparticle protein corona. ACS Nano.

[50] Casals E., Pfaller T., Duschl A., Oostingh G.J., Puntes V.F. (2011). Hardening of the nanoparticle-protein corona in metal (Au, Ag) and oxide (Fe_3_O_4_, CoO, and CeO_2_) nanoparticles. Small.

[51] Loening A.M., Gambhir S.S. (2003). AMIDE: A free software tool for multimodality medical image analysis. Mol. Imaging.

[52] Yokel R.A., Au T.C., MacPhail R., Hardas S.S., Butterfield D.A., Sultana R., Goodman M., Tseng M.T., Dan M., Haghnazar H. (2012). Distribution, elimination, and biopersistence to 90 days of a systemically introduced 30 nm ceria-engineered nanomaterial in rats. Toxicol. Sci..

[53] Mortelé K.J., Segatto E., Ros P.R. (2004). The infected liver: Radiologic-pathologic correlation. Radiographics.

[54] Oró D., Yudina T., Fernández-Varo G., Casals E., Reichenbach V., Casals G., de la Presa B.G., Sandalinas S., Carvajal S., Puntes V. (2016). Cerium oxide nanoparticles reduce steatosis, portal hypertension and display antiinflammatory properties in rats with liver fibrosis. J. Hepatol..

[55] Oostingh G.J., Casals E., Italiani P., Colognato R., Stritzinger R., Ponti J., Pfaller T., Kohl Y., Ooms D., Favilli F. (2011). Problems and challenges in the development and validation of human cell-based assays to determine nanoparticle-induced immunomodulatory effects. Part. Fibre Toxicol..

[56] Yin Q., Yap F.Y., Yin L., Ma L., Zhou Q., Dobrucki L.W., Fan T.M., Gaba R.C., Cheng J. (2013). Poly(iohexol) nanoparticles as contrast agents for in vivo X-ray computed tomography imaging. J. Am. Chem. Soc..

[57] Wason M.S., Colon J., Das S., Seal S., Turkson J., Zhao J., Baker C.H. (2013). Sensitization of pancreatic cancer cells to radiation by cerium oxide nanoparticle-induced ROS production. Nanomedicine.

[58] Sarntinoranont M., Rooney F., Ferrari M. (2003). Interstitial stress and fluid pressure within a growing tumor. Ann. Biomed. Eng..

[59] Mpekris F., Angeli S., Pirentis A.P., Stylianopoulos T. (2015). Stress-mediated progression of solid tumors: Effect of mechanical stress on tissue oxygenation, cancer cell proliferation, and drug delivery. Biomech. Model. Mechanobiol..

[60] Chauhan V.P., Stylianopoulos T., Martin J.D., Popović Z., Chen O., Kamoun W.S., Bawendi M.G., Fukumura D., Jain R.K. (2012). Normalisation of tumour blood vessels improves the delivery of nanomedicines in a size-dependent manner. Nat. Nanotechnol..

[61] Huang R.Y., Neagu M.R., Reardon D.A., Wen P.Y. (2015). Pitfalls in the neuroimaging of glioblastoma in the era of antiangiogenic and immuno/targeted therapy—Detecting illusive disease, defining response. Front. Neurol..

[62] Casals E., Gusta M.F., Piella J., Casals G., Jimenez W., Puntes V. (2017). Intrinsic and Extrinsic Properties Affecting Innate Immune Responses to Nanoparticles: The Case of Cerium Oxide. Front. Immunol..

[63] (2010). Talib Pirmohamed, Janet M Dowding, Sanjay Singh, Brian Wasserman, Eric Heckert, Ajay S Karakoti, Jessica E S King, Sudipta Seal, William T Self Nanoceria exhibit redox state-dependent catalase mimetic activity. Chem. Commun..

