# Supplementary material for: Nanoceria as Safe Contrast Agents for X-ray CT Imaging
Source: Nanomaterials (Basel). 2023 Jul 29;13(15):2208. doi: 10.3390/nano13152208 (PMC10421217; doi:10.3390/nano13152208)
Supplement: Supplementary file 1 [file nanomaterials-13-02208-s001.zip › nanomaterials-2508146-supplementary.pdf]

Supporting Information

Nanoceria as Safe Contrast Agents for X-Ray CT Imaging

Ana García, Juan Antonio Cámara, Ana María Boullosa, Muriel F. Gustà, Laura Mondragón, Simó Schwartz Jr, Eudald Casals, Ibane Abasolo, Neus G. Bastús, Víctor Puntes

1. CeO_2NPs characterization
2. CT contrast values (H.U.) after intravenous injection.
3. Biodistribution of a commercial iodine contrast agent (Iopamidol®-370) injected intravenously.
4. 3D rendering videos after intratumoral injection.
5. ICP-MS analysis of organs after intratumoral injection.
6. Antioxidant tests
7. Photographs of the experimental setup.

1. CeO_2NPs characterization

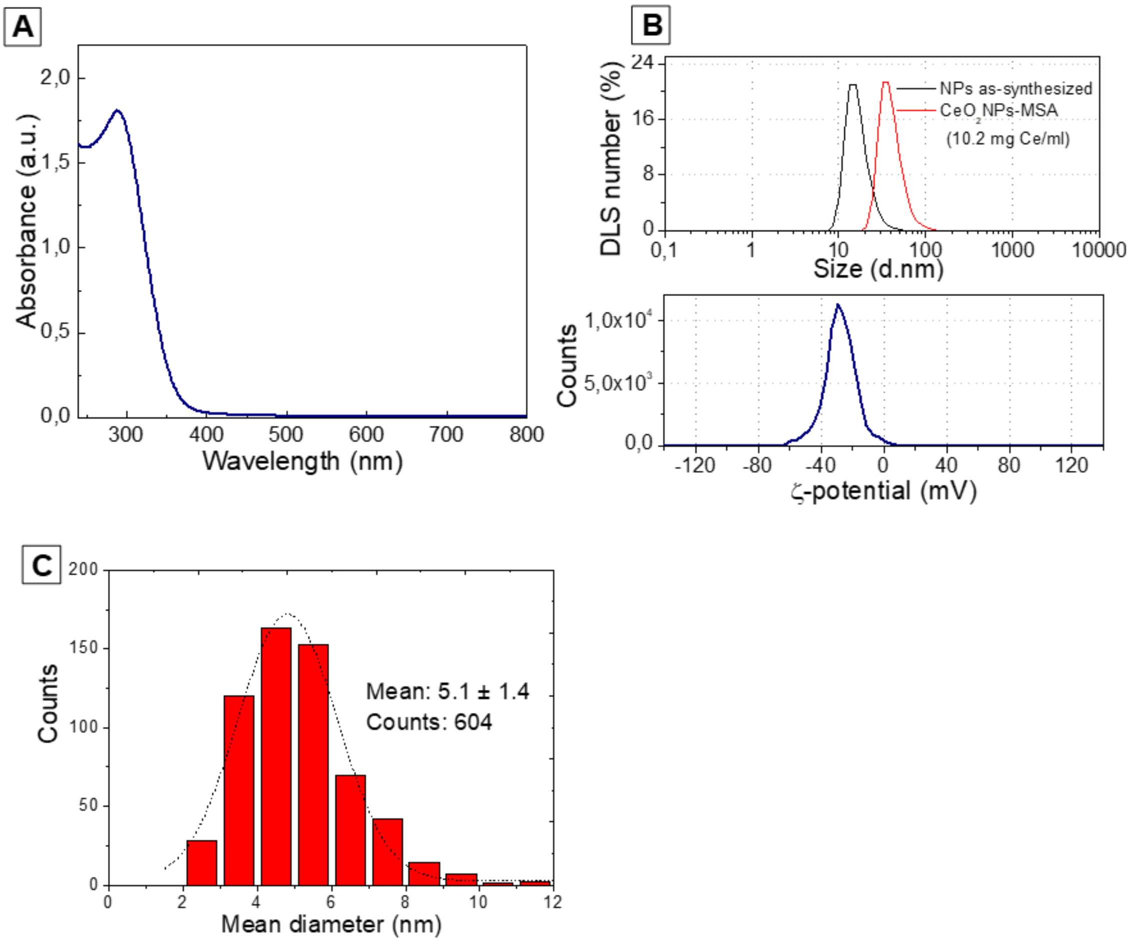

Figure S1. Characterization of CeO_2NPs conjugated with murine serum albumin (MSA) and concentrated up to 10.2mg Ce/ml. **(A)** Corresponding UV-Vis spectra show an absorption peak at 290 nm (sample diluted x20). **(B)** *Top:* Dynamic light scattering (DLS) measurements showing the size distribution profile of CeO_2NPs by Number fits. As-synthesized CeO_2NPs are small aggregates with a number mean of 17.6 nm; once conjugated with albumin and concentrated (10.2 mg Ce/ml), the number mean increases up to 38.7 nm. *Bottom:* ζ-potential profile (-28.2 ± 0.73 mV; media conductivity 0.74 ± 0.02 mS/cm; pH 7.4). Data provided is the mean of three independent measurements. **(C)** **S**ize distribution profile from TEM images indicating a mean diameter of 5.1 ± 1.4 nm.

2. CT contrast values (H.U.) after intravenous injection.

Table S1: CT contrast values (H.U.) corresponding to Figure 2 of the main article. Various regions of interest were measured: spleen, kidney, liver, tumour and sub-lumbar muscle, before CeO_2NPs-MSA injection and at various representative times after injection.

	Pre-injection	*15min*	*30 min*	*1 h*	*2 h*	*24 h*	*7 d*
**Spleen**	80.5 ± 2.1	129.3 ± 4.4	162.7 ± 3.1	164.2 ± 4.7	165.3 ± 7.2	172.2 ± 5.8	155.6 ± 11.8
**Kidney**	45.9 ± 4.8	47.9 ± 3.8	49.0 ± 3.2	48.2 ± 1.0	45.1 ± 2.8	48.8 ± 4.6	38.2 ± 8.0
**Liver**	86.7 ± 2.5	101.7 ± 4.5	115.2 ± 3.9	121.1 ± 5.6	120.0 ± 5.6	121.4 ± 6.9	112.0 ± 13.2
**Tumour**	51.4 ± 1.3	50.2 ± 2.3	53.6 ± 2.1	53.2 ± 2.9	53.5 ± 4.5	50.4 ± 7.7	47.7 ± 6.4
**Muscle**	79.7 ± 1.7	72.1 ± 3.9	74.4 ± 1.7	70.6 ± 0.3	73.9 ± 4.7	76.3 ± 6.7	75.1 ± 4.1

Pre-injection.avi
https://www.dropbox.com/s/di7a65laq1d5s3t/IV_L%20preinyeccion.avi?dl=0

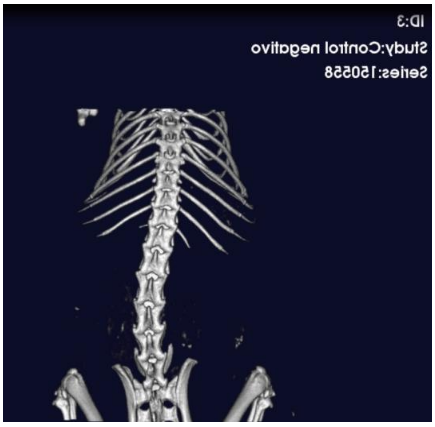

2 h after intravenous injection of CeO_2NPs.avi
https://www.dropbox.com/s/7ouus214r0799k7/IV_L%202%20horas.avi?dl=0

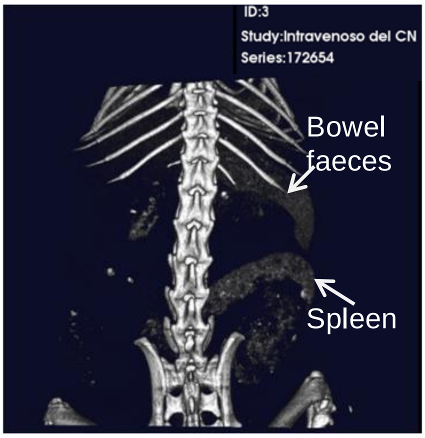

Figure S2. Examples of *in vivo* 3D rendering videos before injection of CeO_2NPs and 2 hours after intravenous injection. Videos were reconstructed from the CT scans studies (512 projections) by using the Quantum F.X. software.

3. Biodistribution of iodine contrast agent injected intravenously.

For comparison, mice were also treated intravenously with a commercial iodine contrast agent (Iopamidol®-370, 150 µl of 175 µg I/ml). The resultant *in vivo* CT coronal images (Fig. S3) indicate that iodine contrast accumulates quickly after injection in the kidneys. 24 hours post-injection there is no trace of it.

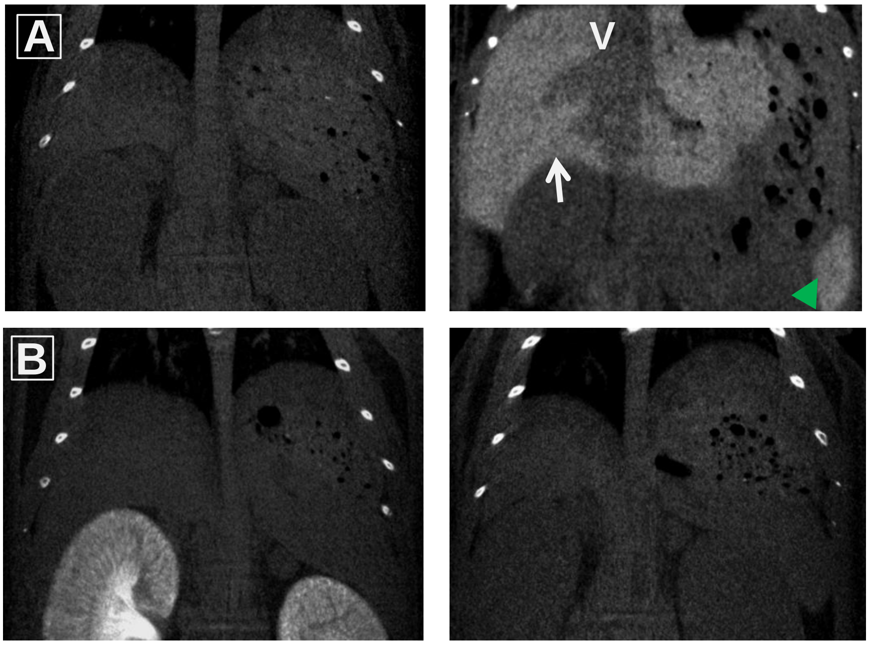

Figure S3. CT coronal images 5 min and 24 h after intravenous injection of CeO_2NPs-MSA (panel A) and the commercial iodine contrast agent Iopamidol®-370 (150 µl of 175 µg I/ml) (panel B). CeO_2NPs accumulate progressively on the liver (white arrow) and spleen (green arrowhead) throughout 24 h after injection, when it can be clearly distinguished the vena cava (V) and the intrahepatic vessels. On the contrary, the iodine contrast is already accumulated in kidneys 5 min post-injection and completely absent 24 h later.

4. 3D-rendering videos after intratumoural injection.

Here it is provided 3D-rendering videos from a mouse bearing a tumour which was intratumourally administered with 70 µl of CeO_2NPs-MSA solution (10.2 mgCe/ml). 3D reconstructions were obtained from the CT scans studies (512 projections) by using the Quantum F.X. software.

15 min after intratumoural injection of CeO_2NPs
https://www.dropbox.com/s/ovitukpnbbwk0yn/Intratumoral%2015min.mpg?dl=0

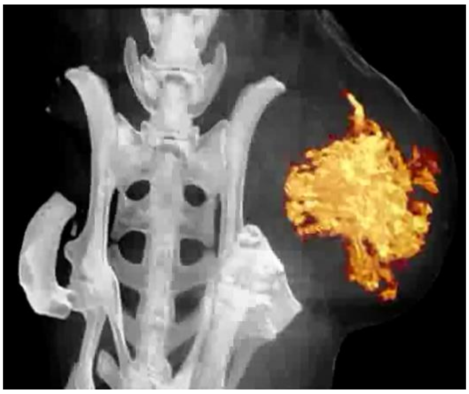

24 h after intratumoural injection of CeO_2NPs
https://www.dropbox.com/s/1oi34m3zfte760r/Intratumoral%20d%C3%ADa%202_Included.avi?dl=0

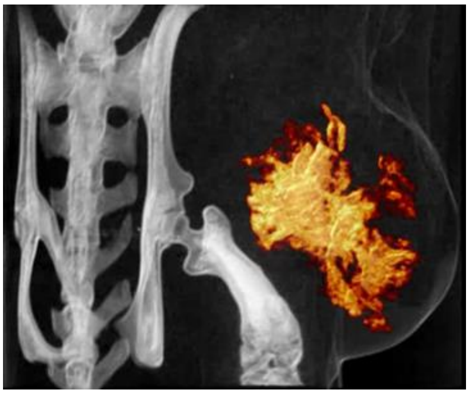

7 days after intratumoural injection of CeO_2NPs
https://www.dropbox.com/s/s5ute5vnslvzcfk/Intratumoral%207%20dias_Included.avi?dl=0

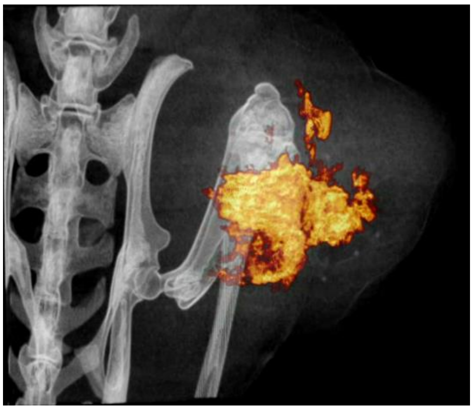

Figure S4. *In vivo* 3D rendering videos and their corresponding snapshots were recorded at 15 min, 24h and 7 days post-intratumoral injection. The videos clearly distinguish the contrast enhancement produced by the CeO_2NPs in the tumour.

5. ICP-MS analysis of organs after intratumoral injection.

After sacrification at day 7, the organs were extracted and analyzed by ICP-MS. Ce was detected primarily on the tumour, whereas no significant amount of Ce was detected in any of the mouse organs or blood plasma. The tumour mass was 1.3 g, which corresponds to a total Ce mass in the tumour of 706.6 ± 35 µg, i.e., 98.9 ± 4.7 % of the injected dose, ID (714 ± 36 µg Ce).

Table S2. ICP-MS analysis of organs after intratumoral injection.

	Ce (µg/organ g)	*% Injected Dose (%ID)*
**Spleen**	< 0.05	< 0.1 %
**Liver**	0.067 ± 0.003	< 0.1 %
**Kidney**	< 0.05	< 0.1 %
**Lung**	< 0.1	< 0.1 %
**Heart**	< 0.1	< 0.1 %
**Brain**	3.5 ± 0.18	0.93 ± 0.05%
**Tumour**	544 ± 27	98.9 ± 4.9 %
**Plasma**	< 1	< 0.1 %

6. Antioxidant tests

The antioxidant properties of current CeO_2NPs-MSA have been tested through the Amplex Red assay (Invitrogen, cat nº A22188), which determines NPs capability to scavenge the representative ROS-type hydrogen peroxide (H_2O_2) (*1*, *2*). Briefly, H_2O_2 aliquots in final concentrations ranging from 0-6 µM were added to the NPssolution (0.5 mg/ml) and let them react for 10 minutes. Then, the Amplex Red reagent was injected, which reacted with H_2O_2 in a 1:1 stoichiometry and produced a red-fluorescent product. The resultant fluorescence emission was measured and indicated the remaining H_2O_2 concentration in each sample.
In Figure S9 it can be seen that CeO_2NPs-MSA are capable of scavenging a significant amount of H_2O_2 compared to control. In particular, H_2O_2 concentration is reduced to ~45% of the initial value. Additionally, to discard that H_2O_2 scavenging is being produced by the surface conjugated albumin (MSA), an identical test was performed on naked CeO_2NPs. As it can be seen in Figure S9, similar results were obtained.

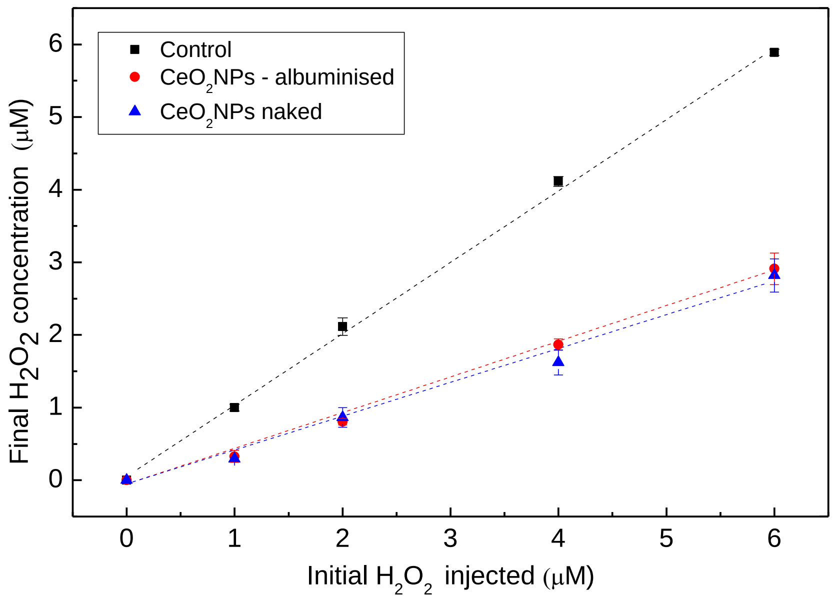

Figure S5. CeO_2NPs-albumin conjugated and naked CeO_2NPs capacity to scavenge hydrogen peroxide (H_2O_2) compared to control, as determined with the Amplex Red test.

7. Photographs of the experimental setup.

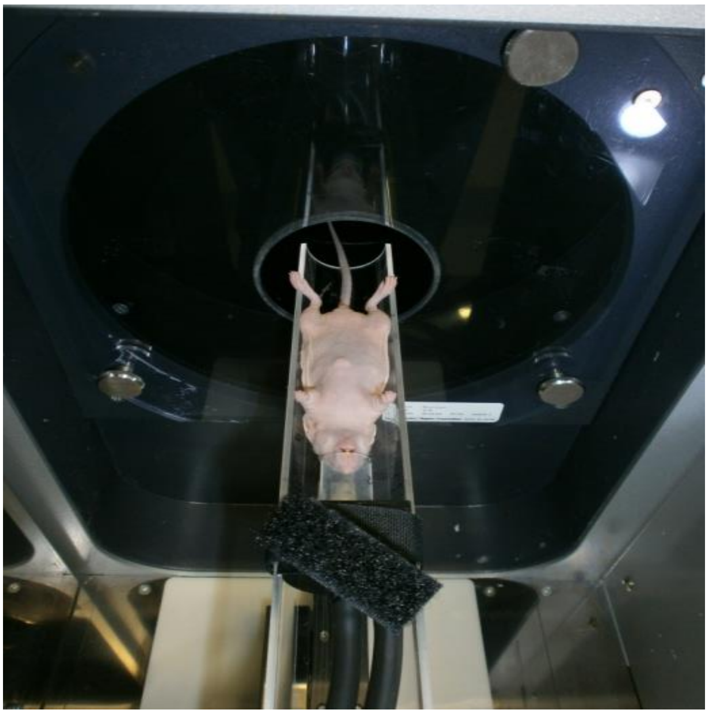

Figure S6. (Left) Mouse anesthetized before a CT scans. (Right) Mouse placed in the plate of the Quantum FX micro-CT instrument (Perkin Elmer, Waltham, MA).

References:

1. T. Pirmohamed *et al.*, Nanoceria exhibit redox state-dependent catalase mimetic activity. *Chem. Commun.* **46**, 2736 (2010).
2. M. S. Wason *et al.*, Sensitization of Pancreatic Cancer Cells to Radiation by Cerium Oxide Nanoparticle-Induced ROS Production, doi:10.1016/j.nano.2012.10.010.
